# Influence of Dielectric Barrier Discharge Treatment on Surface Structure of Polyoxymethylene Fiber and Interfacial Interaction with Cement

**DOI:** 10.3390/ma11101873

**Published:** 2018-10-01

**Authors:** Wei Zhang, Xiao Xu, Fayun Wei, Xueshu Zou, Yu Zhang

**Affiliations:** 1 National and Local Joint Engineering Research Center of Technical Fiber Composites for Safety and Protection, Nantong University, Nantong 226019, China; zhangwei@ntu.edu.cn (W.Z.); z.yu@ntu.edu.cn (Y.Z.); 2 School of Textile and Clothing, Nantong University, Nantong 226019, China; xx18860976127@126.com (X.X.); m18862976230@163.com (X.Z.)

**Keywords:** polyoxymethylene fiber, dielectric barrier discharge, cement, pull-out bonding strength

## Abstract

Polyoxymethylene (POM) fiber was treated with atmospheric dielectric barrier discharge (DBD) plasma to enhance the surface activity of the fiber and interfacial interaction with cement. The physical and chemical properties of samples with different DBD plasma treatment durations were tested and analyzed. Scanning electron microscopy (SEM) and atomic force microscopy (AFM) revealed that the surface roughness of the sample increased significantly as a result of the DBD plasma treatment. Fourier transform infrared spectrophotometer (FTIR) and X-ray photoelectron spectroscopy (XPS) analysis showed that a large number of –COH and –COOH groups were formed on the surface of the sample after DBD plasma treatment. The hydrophilicity of the POM fiber was greatly improved with the increase in the treatment duration. When the treatment duration was longer than 120 s, the fiber surface contact angle decreased from 90° to 43°. The DBD plasma treatment resulted in a decrease in the tensile strength of the POM fiber, but the increase in the amount of –COH and –COOH on the surface of the POM fiber and the increase in the roughness resulted in an increase in the fiber pull-out bonding strength in cement from 2.15 N to 4.68 N.

## 1. Introduction

Polyoxymethylene (POM) is a linear polymer without branches and has a high density and high crystallinity [1,2]. Further, it has excellent features such as good mechanical properties [3], wear resistance, dimensional stability [4,5], chemical resistance, and electrical insulation [6]. In particular, it has good tenacity and outstanding fatigue resistance, so it is often used as reinforcing fibers which can greatly improve the overall performance of the composite materials [7,8]. POM fiber has excellent creep resistance and stress relaxation resistance. Further, it shows no fatigue phenomenon under alternating stress and continuous vibration, so the POM fiber can be used as a fiber-reinforced material for cement mortar, e.g., in constructing bridges, tunnels, dams, and highways [9]. It can reduce the damage caused by the accumulation of stress in these structures. However, the polyether group in the POM fiber has a low polarity and the bonding strength with the cement material is not high. Further, the surface needs to be treated to improve its performance and to improve the interfacial adsorption strength between the fiber and the cement [10].

There are many methods for surface modification of fibers. Plasma surface modification is a dry modification method that does not require water. Moreover, it is fast and has a high efficiency and simple operation. It also causes no pollution and requires less energy than other methods. Further, the modifications only occur on the fiber surface up to 50–100 nm depth, and thus does not change the properties of the fiber matrix [11,12,13]. During plasma treatment, air molecules undergo excitation, ionization, or dissociation, resulting in new excited states, metastable states, free particles, active groups, and various low-temperature plasma active substances such as ions, electrons, and photons [14,15]. It provides abundant active particles and free radicals for chemical reactions. Charged particles, i.e., a mixture of excited atoms and photons, collide with molecules on the substrate surface, causing extensive chemical and physical interactions between the plasma and the fiber surface, such as surface cleaning, physical etching, chemical modification, and polymerization, which can impart new surface characteristics to the fiber [16,17]. Therefore, plasma treatment technology is an effective surface modification technology, and has received more and more attention.

Since POM fiber is an organic material, it differs from cement materials in terms of mechanical, deformation, and physical properties. Upon incorporation of POM fibers into cement, a new interface transition zone is formed, i.e. fiber–matrix interface transition zone. This increases the number of internal defects, expands the stress concentration region, and ultimately affects the mechanical properties and durability of the cement [18,19]. Cement contains a large amount of Ca^2+^ and Mg^2+^ ions, which can undergo chemical interactions with the –OH on the surface of fibers during the hydration process, so that the interface strength of the fiber–cement matrix is greatly improved [20,21]. POM fibers are linear molecules with no polar side groups, and it is difficult to chemically modify their side groups to form –OH structures. Therefore, only the surface of the fibers is treated by plasma technology to form –OH, thus effectively improving the interface strength between the fiber and cement and improving the mechanical properties.

In this study, to improve the interface strength between POM fibers and cement, the surface modification of fibers was performed using the low-temperature air dielectric barrier discharge (DBD) plasma surface-modification technology to study the effect of plasma treatment durations on the surface properties of fibers. The surface morphology and roughness of fibers were characterized by SEM and AFM. The changes in surface chemical groups before and after fiber plasma treatment were analyzed by XPS and FTIR. The hydrophilic POM fiber surface was analyzed by contact angle measurement, and the mechanical properties of the fiber and its pull-out bonding strength in cement were tested.

## 2. Experimental Section

### 2.1. Materials

POM fibers prepared by melt spinning as continuous monofilament with a diameter of 200 μm were supplied by Sobute New Materials Co., Ltd., Nanjing, China. Absolute ethyl alcohol was supplied by Sinopharm Chemical Reagent Co., Ltd., Shanghai, China, and standard Portland cement P.O. 42.5 was obtained from Huaxin Cement Co., Ltd., Nantong, China.

### 2.2. Plasma Treatment and Methods

**Figure 1:**
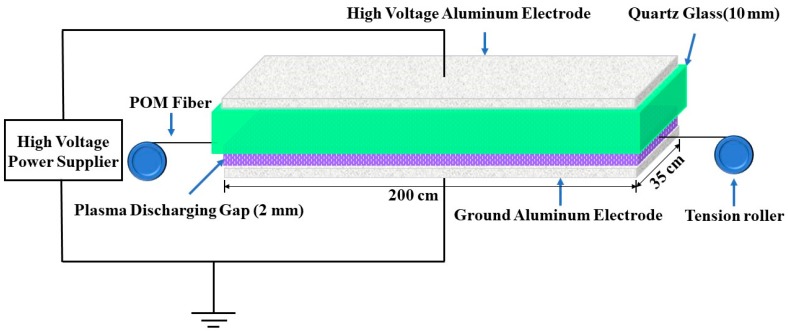
Schematic diagram of DBD plasma treatment equipment.

POM fibers were soaked in absolute ethyl alcohol for 30 min, and then the impurities and residues on the fiber surface were washed away with deionized water. The fibers were put into a vacuum drying oven at 90 °C for 2 h, and were then treated with plasma. All surface modifications of POM fibers were carried out using atmospheric-pressure DBD equipment (APP-350, Institute of Microelectronics of Chinese Academy of Science, Beijing, China). The working mechanism diagram of the DBD plasma treatment equipment is shown in Figure 1.

The DBD plasma was produced between two parallel aluminum electrodes, and the upper aluminum electrode was covered with a 10-mm-thick quartz glass as a dielectric layer. The length and width of the electrodes were 200 cm and 35 cm, respectively. The distance between the quartz glass layer and the ground electrode was 2 mm. The tension rollers were used to keep the fiber straight. The fibers were kept stationary during the whole treatment. After the treatment, the fibers were quickly removed by turning the roller and wrapped around the surface of the roller. The plasma treatment power was 300 W, an air atmosphere was employed, and the treatment duration was changed between 30 s and 180 s. 

The changes in the surface morphology of POM fibers as a result of DBD plasma treatment were analyzed by Hitachi S4800 SEM (Hitachi, Tokyo, Japan). All samples were sprayed with gold and observed under a working voltage of 3 kV. Besides, the surface modifications were also investigated using a Bruker Dimension Edge AFM (Bruker, Billerica, MA, America). The 5 μm × 5 μm scans were recorded in the tapping mode, and the surface average roughness was analyzed.

The surface functional groups of POM fibers were analyzed by FTIR spectra recorded on Nicolet iS10 (Thermo Scientific, Waltham, MA, America) over the wavenumber range from 400 to 4000 cm^−1^. The attenuated total reflection (ATR) spectra was recorded at 0.4 cm^−1^ resolution and using 32 scanning times. The chemical composition of the fiber surface was analyzed using Escalab 250Xi XPS (Thermo Scientific). The X-ray radiation source was Al K_α_ radiation (1486.6 eV) with an operating power of 150 W, and the spectra were taken at 90°. The pass energies for the survey scan and C1 s scan were 160 eV and 40 eV, respectively. 

The water contact angle was measured by a JC2000 C2 Contact Angle Analyzer (Zhongchen Digital Technic Apparatus Co., Ltd., Shanghai, China). Deionized water was sprayed as a mist on the fiber surface by the sprayer. The contact angle was recorded after the droplets stabilized, and the average value was obtained from 15 measurements.

The tensile strength and pull-out bonding strength of the POM fibers were studied using the Instron 5696 universal testing machine (Instron, Norwood, MA, America). The collet spacing was 5 cm and the stretching speed was 100 mm/min. A plastic mold made from polymethyl methacrylate (PMMA) was used to prepare the specimen for the pull-out test, and the mold dimensions were 10 cm × 1 cm × 0.5 cm (L × W × H) [22,23]. Subsequently, the cement paste prepared with water/cement ratio of 0.35 was carefully poured into the mold from the space between ten staple fibers which were separated by 1 cm distance. Then, vibration was performed to form a uniform cement matrix around the fibers. After one day of curing, cutting was performed and demolded to obtain a pull-out test sample of 1 cm × 1 cm × 0.5 cm. Then, the samples were cured in a standard moist room at the temperature of 20 °C and relative humidity of 95% for seven days.

## 3. Results and Discussion

### 3.1. Surface Morphology Analysis

**Figure 2:**
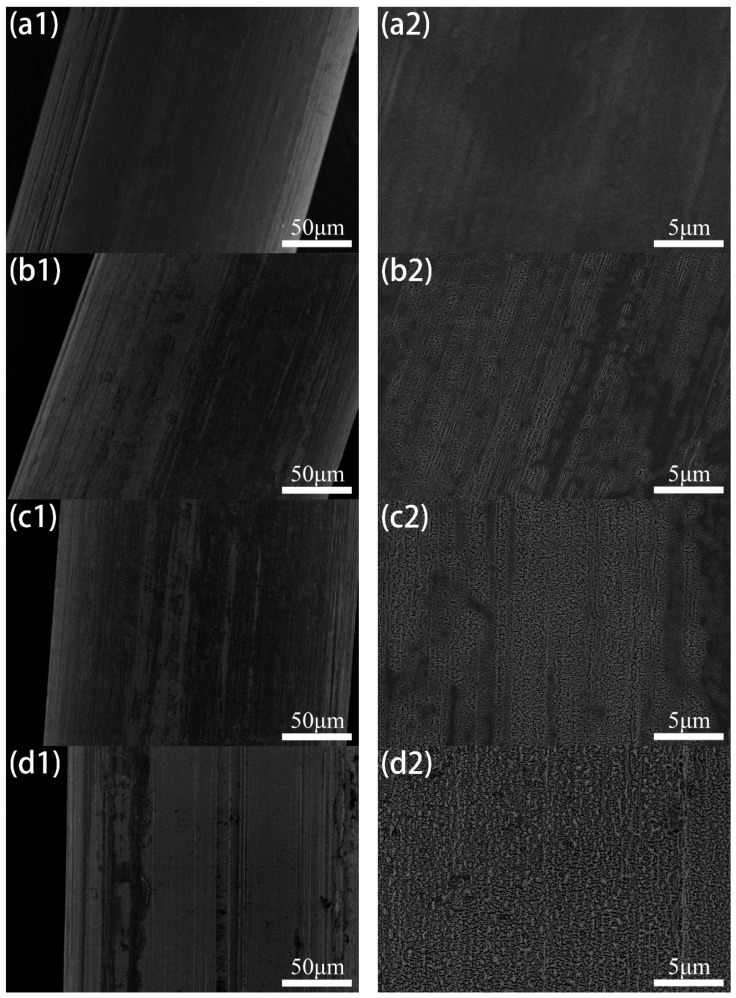

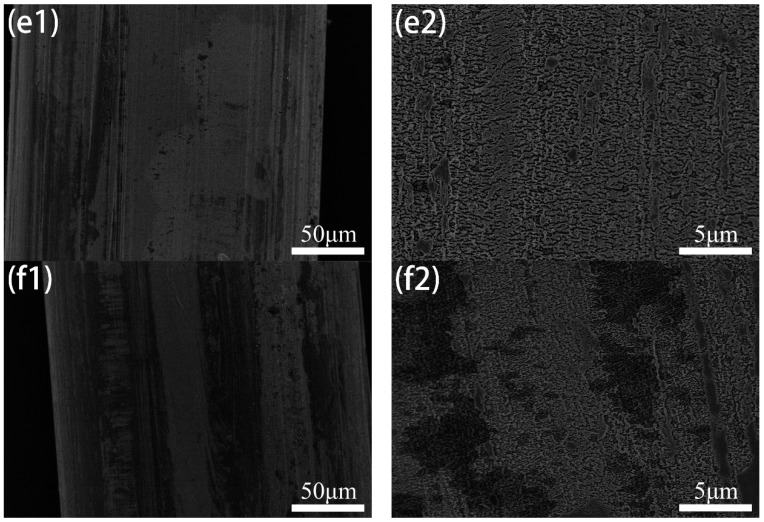
SEM images: of untreated POM fiber (**a1**,**a2**); and fiber subjected to DBD plasma treatment for: 30 s (**b1**,**b2**); 60 s (**c1**,**c2**); 90 s (**d1**,**d2**); 120 s (**e1**,**e2**); and 180 s (**f1**,**f2**).

SEM images of POM fiber with different plasma treatment durations are shown in Figure 2. It can be seen that the surface of the untreated fibers is relatively smooth, but there are longitudinal stripes and uneven protrusions. Etching of varying degrees appears on the surface of the fiber after plasma treatment. The longer is the treatment duration, the larger is the surface etching cave [24,25]. When the fiber was treated with the DBD plasma for 30 s, a small number of cracks and irregular holes appeared on the surface of the fiber. As the treatment duration increased, the number of cracks and holes gradually increased. When the treatment duration reached 90 s, cracks and holes were spread over the surface of the fiber. With the further increase of treatment durations, it was found that the surface of the fiber was a little sticky. That means the fiber surface has partially melted.

**Figure 3:**
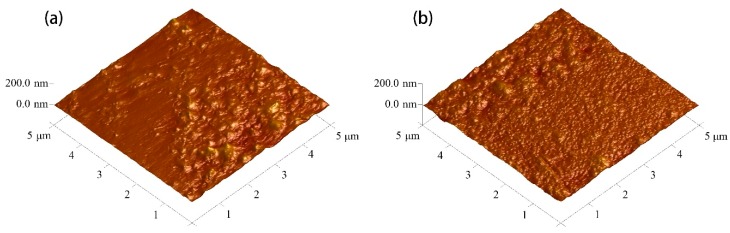

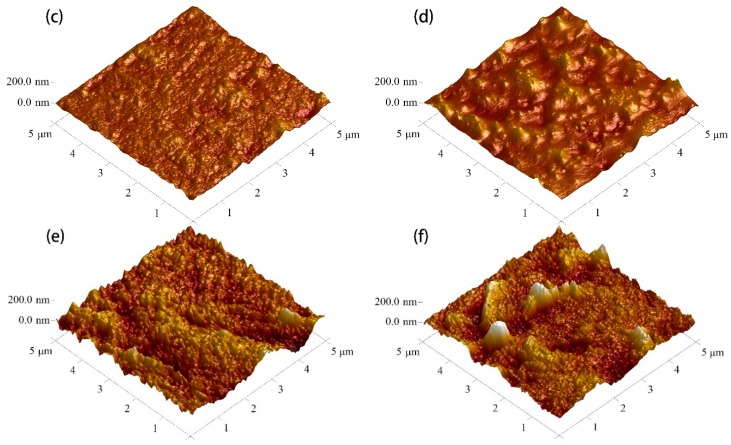
AFM images of: untreated POM fiber (**a**); and fiber subjected to DBD plasma treatment for: 30 s (**b**); 60 s (**c**); 90 s (**d**); 120 s (**e**); and 180 s (**f**).

The three-dimensional AFM images of POM fiber before and after DBD plasma treatment are presented in Figure 3, and the obtained surface average roughness (R_a_) and the root mean square roughness (R_ms_) are shown in Table 1. The results show that the surface morphology of fibers changed significantly as a result of the DBD plasma treatment. The surface of the untreated fibers was relatively smooth, and the values of R_ms_ and Ra were 7.38 and 5.72 nm, respectively. With the treatment durations increasing, the roughness of all samples increased significantly. When the plasma treatment duration was 180 s, R_ms_ and R_a_ of the treated fibers increased to 14.20 nm and 10.40 nm, respectively. This indicated that the surface roughness of fibers could be effectively controlled by adjusting the DBD plasma treatment duration.

In DBD plasma treatment, the energy is sufficient to destroy the chemical bond energy in the fiber [26,27]. The etching of the fiber surface intensified as the treatment duration increased, which means some of the chemical bonds on the surface of the fiber may be broken, resulting in new surface characteristics. At the same time, the oxygen plasma has a filamentous discharge phenomenon, causing thermal abrasion, evaporation, degradation, and other processes on the surface of the fiber, and some structures are oxidized and removed, resulting in remarkably increased surface roughness [28,29]. When the DBD plasma treatment duration continued to increase to more than 120 s, the surface of the fiber began to fuse a little bit, which affected the surface roughness of the fiber to a certain extent. In general, with the increase of plasma treatment duration, the surface roughness of treated fiber is higher than that of untreated fiber.

### 3.2. Chemical Analysis of Surface

**Figure 4:**
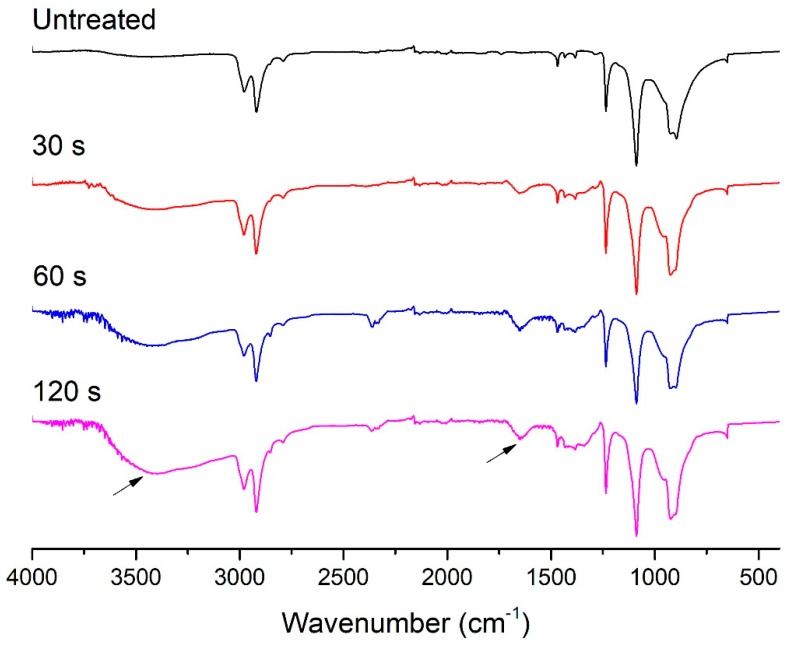
FTIR spectrum of untreated POM fiber and fiber subjected to DBD plasma treatment for different durations.

The FTIR spectrum of the POM fiber before and after DBD plasma treatment is shown in Figure 4. The double peaks at 900 cm^−1^ and 935 cm^−1^ in the figure are the combined peaks corresponding to the stretching vibration of –C–O–C– and the rocking vibration of –CH_2_–, respectively, which are the characteristic spectra of POM [30]. It can be seen in Figure 4 that, as the DBD plasma treatment duration increased, the characteristic absorption peak intensity increased significantly. This indicates that the plasma bombardment destroys the chemical structure (such as –C–O–C– and –CH_2_–) of the fiber surface during plasma treatment. The plasma-treated samples all showed a weak conjugated carbonyl –C=O absorption peak at a wavelength of about 1650 cm^−1^, and a typical hydrogen bonding –OH peak appeared between 3300 and 3500 cm^−1^, indicating that the DBD plasma treatment results in the appearance of a certain number of hydrophilic groups such as –C–OH and –COOH on the surface of the fiber.

**Figure 5:**
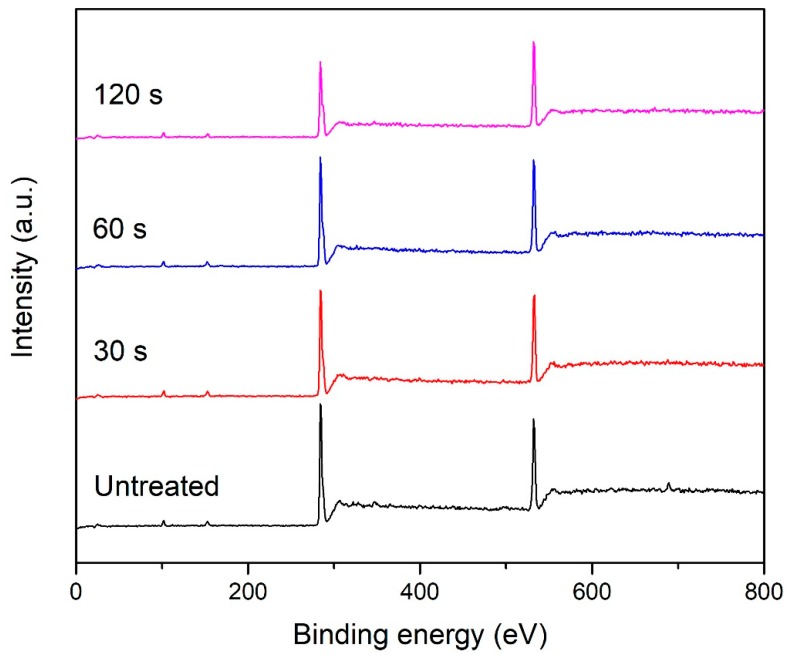
XPS survey spectrum of untreated POM fiber and fiber subjected to DBD plasma treatment for different durations.

XPS survey spectra of the POM fiber before and after DBD plasma treatment are shown in Figure 5. Two strong electron peaks at 285 eV and 532 eV in the spectrum are C1s and O1s peaks, respectively, and the N1s peak at 400 eV is too small to show. Nitrogen was present in a very small amount before and after DBD plasma treatment, and the content remained almost the same, indicating that the nitrogen in the air did not participate in the reaction during the plasma treatment. Table 2 shows the relative chemical compositions and atomic ratios of fibers before and after DBD plasma treatment as measured by XPS. The POM fiber is mainly composed of three elements, namely, carbon, oxygen, and hydrogen, and the proportions of carbon and oxygen are 78.72% and 20.13%, respectively. As the treatment duration increases, the content of oxygen increased gradually, and the content of carbon decreased in contrast. When the DBD plasma treatment duration was 120 s, the proportion of oxygen atoms increased to 26.38%, and the proportion of carbon elements decreased to 72.70%. In the DBD plasma treatment, the oxygen in the air is ionized, resulting in a high-energy excited state, and it reacts with the activated carbon atoms and active hydrogen atoms generated by the bombardment of –CH or –COC– bonds on the surface of the fiber to form new –COH or –COOH bonds.

**Figure 6:**
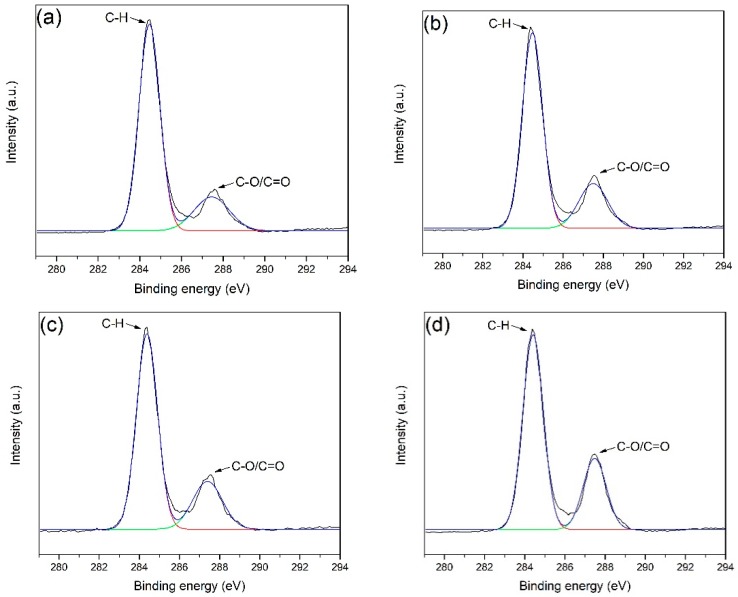
XPS spectra deconvolution of: the C1s peak of untreated POM fiber (**a**); and fiber subjected to DBD plasma treatment for: 30 s (**b**); 60 s (**c**); and 120 s (**d**).

To study the change in the surface functional groups of POM fibers after the DBD plasma treatment, the C1s peak obtained by the XPS scan was treated by peak separation, and two sub-peaks, as shown in Figure 6, were obtained. The sub-peak at 284.6 eV is the C1s peak assigned to –CH and the one at 287.5 eV is the C1s peak corresponding to C–O or C=O [31,32,33]. The results of peak separation before and after DBD plasma treatment and the changes in the surface groups of fibers are presented in Table 3. The data show that the number of –CH bonds on the surface of the fiber is significantly reduced by plasma treatment, while the number of C–O and C=O bonds is significantly increased. Combined with the previous test analysis results, it can be inferred that the DBD plasma treatment results in the formation of polar groups such as carbonyl group, carboxyl group, and hydroxyl group on the surface of the fiber.

### 3.3. Hydrophilic Analysis of Surface

**Figure 7:**
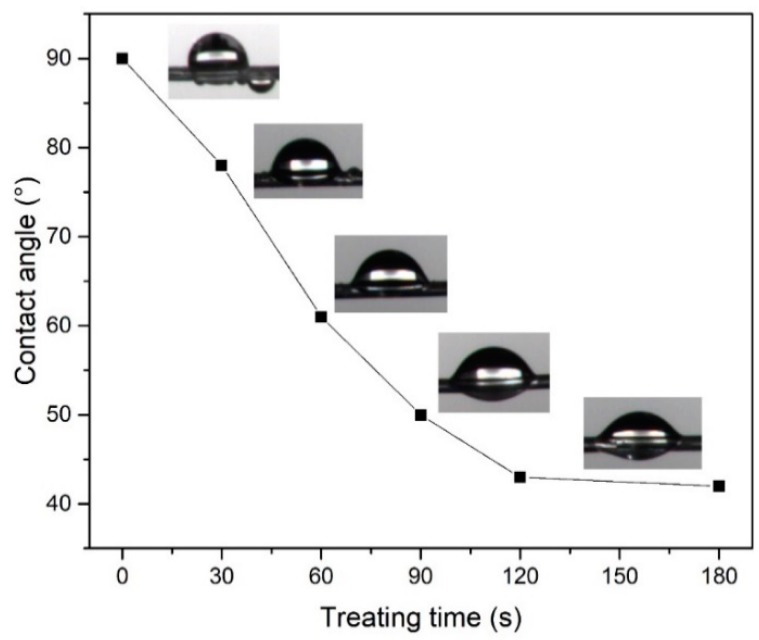
Influence of DBD plasma treatment duration on contact angle.

The relationship between the contact angle of deionized water and the plasma treatment duration is shown in Figure 7. The results show that the contact angle of the untreated POM fibers was 90°, and the contact angle was significantly lowered after the DBD plasma treatment. When the plasma treatment duration was 30 s, the contact angle became 78°, and when the plasma treatment duration was increased to 120°, the contact angle decreased to 43°, indicating that a large number of hydrophilic groups appeared on the surface of the material as the plasma treatment duration increased [34,35]. As shown in Figure 6, the XPS spectra indicate that the –CH bond transformed into –COH or –COOH, so the hydrophilicity of the fiber surface improved greatly. However, as the DBD plasma treatment duration increased, the contact angle did not decrease further; for example, it was 43° at 120 s and 42° at 180 s. This is due to the limited oxygen content in the air. As the treatment duration increases, the –COH or –COOH content does not continue to increase, and the degradation of the POM molecular chain increases.

### 3.4. Tensile Properties of Fiber

**Figure 8:**
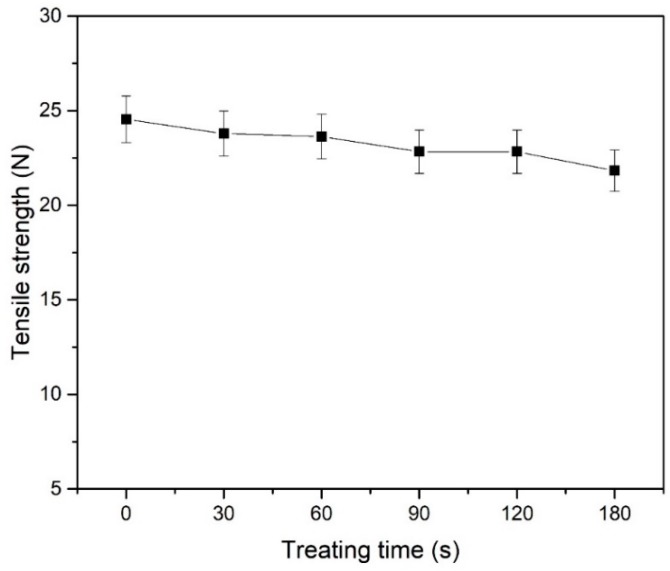
Influence of DBD plasma treatment duration on tensile strength.

The effect of treatment duration on the tensile strength of POM fiber is shown in Figure 8. The mechanical properties of the fibers deteriorated with increasing treatment duration. When the treatment duration is less than 180 s, the tensile strength of the fiber decreased from 24.54 N to 21.83 N, and the strength loss rate reached 11.04%. This is because the DBD plasma treatment causes a large amount of etching and results in the formation of micropores on the surface of the fiber; as a result, the fiber strength decreases, thus, as the plasma treatment duration increases, the mechanical properties of the fiber deteriorate. However, since the POM fiber selected for this experiment has a large diameter of 200 μm, the surface nanoscale etching and micropores do not have a substantial influence on the mechanical properties of the fiber. Thus, after plasma treatment, the mechanical properties of the fiber remain suitable for application in cement.

### 3.5. Pull-Out Bonding Strength Analysis

**Figure 9:**
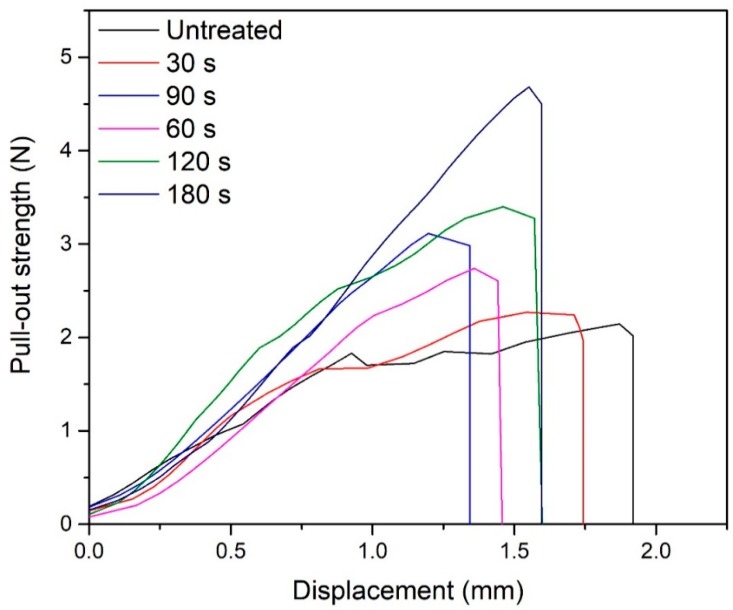
Pull-out bonding strength of DBD plasma-treated POM fibers for different durations.

**Figure 10:**
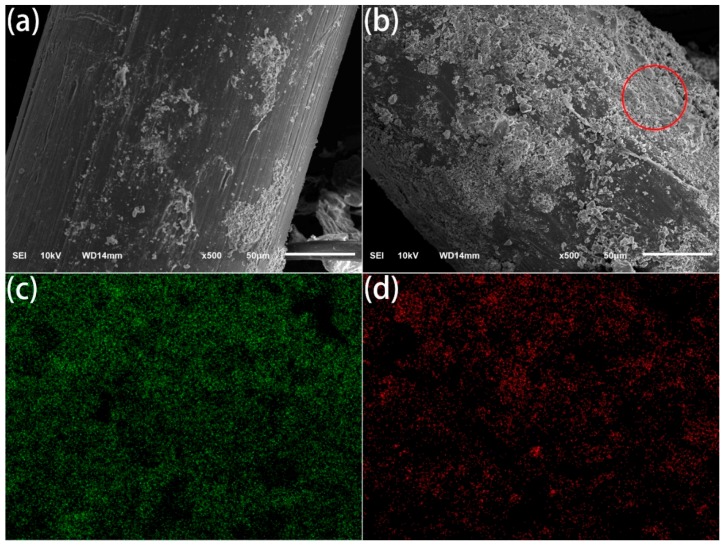
SEM photos of untreated (**a**) and treated for: 180 s (**b**) POM fibers; and EDS images of Ca (**c**) and Mg (**d**).

The pull-out bonding strength of POM fibers for different durations is shown in Figure 9. The pull-out bonding strength of the fiber in cement mainly depends on the interaction between the fiber and the cement interface layer, and its pull-out bonding strength is much smaller than the tensile strength of the fiber itself. The results show that, with the increase in the DBD plasma treatment duration, the pull-out bonding strength of the fiber in the cement increases significantly. When the plasma treatment duration was 180 s, the pull-out bonding strength of the fiber increased to 4.68 N from 2.15 N. There are two main reasons for the increase in the pull-out bonding strength. On the one hand, as the plasma treatment duration is increased, the surface roughness of the fiber increases; therefore, the friction coefficient between the cement and the fiber increases, thus the pull-out bonding strength of the fiber in cement is increased. On the other hand, as the treatment duration increases, the amount of –OH and –COOH groups on the surface of fiber increase. In the hydration process of cement, a large amount of ions, such as Ca^2+^ and Mg^2+^, can interact with the –OH and –COOH of fiber surface, which is beneficial to improve the interface strength between the fiber and cement. The SEM photos of interfacial transition zone on pull-out fibers are shown in Figure 10a,b. Obviously, the residual cement on the surface of untreated fiber is very few. On the contrary, the treated fiber surface has more coagulation residues which are evenly distributed. EDS detection was carried out for the circle area on plasma treated fiber surface, and the results showed that there were a lot of calcium and magnesium in this area, as shown in Figure 10c,d. These prove that the –OH and –COOH groups on the surface of the plasma treated fibers interact with Ca^2+^ and Mg^2+^ in the cement, which is enhancing the interfacial strength between the fibers and cement. In addition, after the plasma treatment, the POM fiber modulus increased, and it can withstand larger load under a small strain. After the cement material is subjected to the same stress, the incorporation of plasma treated fiber can prevent crack generation and propagation more effectively than untreated fiber.

## 4. Conclusions

In this study, the effects of the DBD plasma treatment duration on the properties of POM fiber, such as surface morphology, chemical analysis, hydrophilicity, tensile strength, pull-out bonding strength of the fibers in cement, and EDS of interfacial transition zone were studied. Fibers with different surface roughness can be obtained by controlling the DBD plasma treatment duration. The DBD plasma treatments result in a decrease in the amount of –CH groups on the surface of the fiber, an increase in the amount of the –COH and –COOH groups, and an increase in the hydrophilicity of the surface of the fiber. The hydrophilicity of the fiber greatly increased and the tensile strength of the fibers decreased as the DBD plasma treatment duration increased. However, due to the increase in the amount of –OH and –COOH on the surface of the fiber and the increase in the roughness, the pull-out bonding strength of the fiber in the cement significantly increased, which will prevent the generation and propagation of the cement cracks more effectively.

## Figures and Tables

**Table 1:** Surface roughness of POM fiber before and after the DBD plasma treatment.

Condition		R_ms_ Roughness (nm)	R_a_ Roughness (nm)
Untreated		7.38	5.72
Plasma treatment for	30 s	7.40	5.47
	60 s	13.10	9.31
	90 s	15.30	10.10
	120 s	12.50	9.97
	180 s	14.20	10.40

**Table 2:** Relative chemical composition and atomic ratios determined by XPS for untreated POM fiber and for fiber after DBD plasma treatment.

Condition	Chemical Composition (%)			Atomic Ratio (%)	
	C1s	O1s	N1s	O/C	N/C
Untreated	78.72	20.13	1.15	0.26	0.01
30 s Plasma treatment	77.10	21.85	1.05	0.28	0.01
60 s Plasma treatment	76.39	22.71	0.90	0.30	0.01
120 s Plasma treatment	72.70	26.38	0.93	0.36	0.01

**Table 3:** Results of deconvolution analysis of C1s peaks for untreated POM fiber and fiber after DBD plasma treatment.

Condition	Relative Area Corresponding to Different Chemical Bonds (%)	
	C–H	C–O/C=O
Untreated	78.51	21.49
30 s Treated	75.46	24.54
60 s Treated	74.50	25.50
120 s Treated	70.99	29.01
